# Electrophysiological Studies of Cognitive Reappraisal Success and Failure in aMCI

**DOI:** 10.3390/brainsci11070855

**Published:** 2021-06-27

**Authors:** Shasha Xiao, Yingjie Li, Meng Liu, Yunxia Li

**Affiliations:** 1 School of Communication and Information Engineering, Shanghai Institute for Advanced Communication and Data Science, Shanghai University, Shanghai 200444, China; sansa@shu.edu.cn; 2 Department of Neurology, Tongji Hospital, School of Medicine, Tongji University, Shanghai 200092, China; 1811307@tongji.edu.cn

**Keywords:** mild cognitive impairment, emotion regulation, cognitive reappraisal, event-related potential, late positive potential, theta power

## Abstract

Background: Although successful reappraisal relies on cognitive resources, how cognitive impairment affects brain processes related to cognitive reappraisal is not yet clear. Methods: Forty-four amnestic mild cognitive impairment (aMCI) subjects and 72 healthy elderly controls (HECs) were divided into the MCI-Failure (*n* = 23), MCI-Success (*n* = 21), HEC-Failure (*n* = 26), and HEC-Success (*n* = 46) groups according to changes in self-reported affect using reappraisal. All participants viewed 30 negative and 30 neutral images preceded by straightforward descriptions of these images and 30 negative images preceded by more neutral descriptions. Results: Reappraisal failure was found to be more common in people with MCI. Reappraisal failure is associated with altered neurophysiological indices of negative-reappraisal stimuli processing that are reflected in smaller theta responsivity to negative-reappraisal stimuli between 350–550 ms. The MCI-Success group showed enhanced LPP for negative-reappraisal stimuli from 1200 to 3500 ms, reflecting compensatory effort to complete the reappraisal task, while subjects in other groups showed reduced LPP for negative-reappraisal stimuli from 550 to 1200 ms. Conclusions: These findings deepen our understanding of how cognitive decline impacts reappraisal and informs early diagnosis and interventions for MCI.

## 1. Introduction

Amnestic mild cognitive impairment (aMCI) is a dominant subtype of MCI in which memory loss is the predominant symptom, accounting for 66.5% of all MCI cases [1]. People with aMCI are more likely than other subtypes to develop dementia, which is considered a precursor to Alzheimer’s disease (AD) [2,3,4]. Since there is still no effective treatment for AD [5], early diagnosis of MCI plays a vital role in the early intervention and treatment of Alzheimer’s disease. Affective and emotion dysregulation symptoms such as anxiety and depression are common in people with MCI [6,7] and are accompanied by other neuropsychiatric symptoms correlated with poorer overall outcomes [8] and a higher risk for dementia conversion [9]. Therefore, investigating emotion regulation in MCI can provide helpful information for the early diagnosis and intervention of MCI.

Converging evidence has suggested that the emotional regulation ability of healthy elderly individuals remains stable or even improves. For example, older adults can control their emotion better than younger adults [10,11] and can maintain a stable mood for a long time [12]. This phenomenon may be associated with a “positive effect” on older people’s attention and memory [13,14]. The emotional effect on memory was found to be impaired in AD patients [15] and relatively well preserved for higher-performing MCI than for lower-performing MCI subjects [16]. Psychological studies revealed that MCI participants endorsed significantly greater maladaptive emotion regulation strategy use than healthy elderly participants [17]. Although emotion regulation has been widely investigated, the physiological responses and neural substrates of emotion regulation in people with MCI using specific regulation strategies remain unclear.

Cognitive reappraisal is an effective emotion regulation strategy in which one reinterprets the meaning of emotion-eliciting situations [18]. Compared with distraction, cognitive reappraisal has a lasting effect [19] and does not have side effects on memory compared with suppression [20,21]. Meanwhile, the use of reappraisal increases with age [22]. Therefore, we used cognitive reappraisal as the emotion regulation strategy to better understand emotional dysregulation in people with MCI. Many fMRI studies have explored the brain regions involved in cognitive reappraisal [23,24]. Morawetz et al. (2020) identified four large-scale neural networks underlying emotion regulation through meta-analysis and cluster analysis. They found a frontoparietal network and a left-lateralized prefrontal network, linked mainly to the cognition domain [25]. Previous studies have shown that despite memory decline, the executive function is also impaired in aMCI [26,27], which may affect the efficiency and time course of emotion regulation. Combined with these results, the effectiveness of downregulating emotions will evidently be influenced by cognitive decline, but this influence has not yet been systematically tested. The influence of cognitive decline on emotion regulation has been investigated by comparing younger and older adults [28,29]. For example, by analyzing behavioral and fMRI data, Opitz et al. found that healthy older adults reported a smaller reduction of unpleasantness compared to younger adults using reappraisal (*p* = 0.06). They showed hypoactivation relative to younger adults in the ventral lateral prefrontal cortex (vlPFC) either in down- or upregulating emotion [30]. These results indicated that the emotional regulation ability of the elderly is related to the extent of cognitive resources that can be recruited. However, to date, limited studies have investigated cognitive reappraisal in people with MCI.

Emotion regulation is well known to be a psychological process that changes over time, and EEG can distinguish the different stages of emotion regulation processing. Theta-band oscillations have been found to be critical for synchronization between cortical and limbic regions during the processing of emotional stimuli [31]. For example, EEG theta (4–8 Hz) power can be enhanced by emotional stimuli at various scalp locations. Posterior theta enhancement has been associated with the increased perceptual processing driven by affective attention [32,33]. This emotional effect may be because theta band oscillations underpin large-scale synchronization across sensory brain areas and other cortical regions, such as frontoparietal attention areas [34,35]. Some studies have reported two early theta power peaks (occurring before and after approximately 300 ms) that were enhanced by emotional stimuli, of which the first peak was not affected by attention, and the second peak was influenced by attention [36,37,38]. Late positive potential (LPP), a central-parietal event-related potential, has been extensively investigated in emotion regulation [39]. LPP is sensitive to both emotional stimuli and cognitive reappraisal modulation [40,41,42]. Most studies found an enhanced LPP for negative stimuli and a reduced LPP for the downregulation of negative stimuli using cognitive reappraisal [43,44,45,46]. However, some researchers reported an enhancement of LPP as an index of reappraisal success [47,48,49]. For example, Langeslag and Surti [49] found that the LPP was increased by downregulating responses to high arousal images but was not significantly affected by downregulating responses to low arousal pictures. Cao et al. [50] divided young subjects into a reappraisal success group and a failure group based on their behavioral performance of reappraisal and found different dynamics of LPP during reappraisal. According to Cao’s study, to characterize the difference between MCI and HEC using reappraisal, it is better to consider their behavioral performance. Therefore, MCIs and HECs were divided into subgroups of reappraisal success and failure groups based on whether they successfully decreased their unpleasant feelings to negative pictures using reappraisal. In addition, it is still unclear how cognitive impairment affects theta power and LPP during a cognitive reappraisal task. Therefore, this study used these theta power and LPP measurements as objective electrophysiological correlates of emotion perception and regulation and investigated the differences between MCI and healthy elderly controls.

This study aimed to investigate whether cognitive decline affects reappraisal in people with MCI and how. There were three hypotheses: (a) MCI subjects would show greater decreased reappraisal ability than healthy elderly subjects in both success and failure groups from the behavioral perspective; (b) MCI subjects would show distinct theta power and LPP characteristics compared to healthy older adults during reappraisal.

## 2. Materials and Methods

### 2.1. Recruitment, Inclusion Criteria, and Participants

MCI subjects were recruited from the Department of Neurology and the Department of Memory Clinic of Shanghai Tongji Hospital, and sex-, age-, and education-matched healthy elderly controls (HECs) were recruited from the local community. The people with MCI underwent a standardized diagnostic program including a full physical and neurological examination, MRI scan or cranial CT, laboratory screening for treponema pallidum, vitamin B12, free tetraiodothyronine (FT4), thyroid function (free triiodothyronine (FT3), folic acid, thyroid-stimulating hormone (TSH)), and HIV antibodies, as well as cognitive screening, i.e., Mini-Mental State Examination (MMSE), the Clinical Dementia Rating scale (CDR) [51], and the Hachinski Ischemic Score (HIS) [52]. The Hamilton Anxiety Rating Scale (HAMA) [53] and the Hamilton Depression Rating Scale (HAMD) [54] were used to evaluate their depressive symptoms and their severity. In addition, memory function, language function, executive function, and visual space navigation function were measured using neuropsychological tests (see Supplemental Materials for details of the tests).

Participants with dementia, mental illness, history of stroke, or Parkinson’s disease were excluded. According to pre-existing criteria for MCI [55], MCI was designated by a neurologist based on all examination results. Specifically, MCI was diagnosed when (1) Clinical Dementia Rating (CDR) = 0.5 and (2) no less than two cognitive domains were impaired or no less than two cognitive tests within the same cognitive domain scored below cutoffs (see Supplementary Materials for the details of the exclusion criteria). In total, we included 44 aMCI and 72 HEC subjects who were Chinese, between 55 and 86 years of age, and not taking medicines such as antidepressants, cholinesterase inhibitors, or hypnotics. Ethical approval was granted by the Ethics Committee of Tongji Hospital, and all participants provided written informed consent. Some of the datasets have also been used for another study using ERP and sLORETA analyses, but MCI and HEC subjects were not divided into subgroups considering their behavioral performance (under review).

### 2.2. Stimuli and Procedure

**Figure 1:**
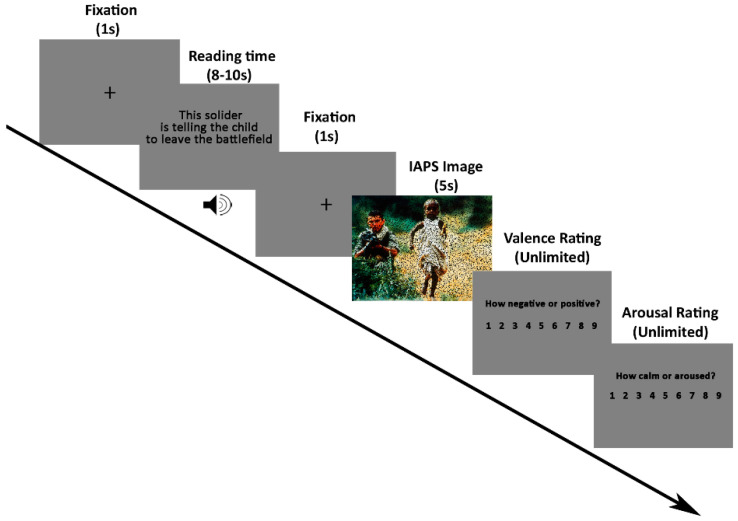
Schematic representation of the cognitive reappraisal task. A black fixation cross was presented in the centre of a grey screen for one second, then a neutral or negative description of the upcoming picture appeared. The descriptions were read out (3–5 s), and when the sound was over, there was a reading time of more than 5 s for the subjects to comprehend its meaning. After reading, a black fixation cross was presented on a black screen for 1 s, telling subjects that reading time was over and attracting their attention to the centre of the screen. Then, a picture (either negative or neutral) was displayed for 5 s. Following each picture, participants were asked to rate each picture on valence (1 = extremely negative, 9 = highly positive) and arousal (1 = calm, 9 = aroused), separately. Note: For copyright reasons, we blurred the image, but they were showed clearly to participants.

In the current study, we used the cognitive reappraisal paradigm described in Foti and Hajcak [45]. Figure 1 displays the procedures. Each trial began with a black fixation cross on a grey screen (1 s) followed by a sentence (5 s reading time after an older adult read it out), followed in turn by an unpleasant or neutral picture shown for 5 s. Upon image offset, participants were required to rate each picture on valence (1 = extremely negative, 9 = highly positive) and arousal (1 = calm, 9 = aroused) using the Self-Assessment Manikin [56]. Ninety trials were presented in five blocks of eighteen, each containing 12 negative images and 6 neutral images. Half of the negative pictures were preceded by negative descriptions about the pictures (negative-watching condition, Neg), and the other half were preceded by more neutral descriptions (negative-reappraisal condition, Rea). All neutral pictures were preceded by neutral descriptions about the pictures (neutral-view condition, Neut). The order of trials was randomized in each block for each subject. Participants were told to look at all the pictures and understand them as described in the preceding sentence. Ninety pictures (60 negative and 30 neutral images) were selected from the International Affective Picture System (IAPS) [57]. Seventy-five out of ninety images were the same as the images used in Foti and Hajcak [45]. Negative and neutral pictures differed on normative ratings of valence (M = 2.94, SD = 0.74 for negative; M = 5.10, SD = 0.40 for neutral) and arousal ratings (M = 5.54, SD = 0.86 for negative; M = 2.94, SD = 0.73 for neutral). Participants went through practice trials to fully understand the experimental procedure and the meaning of valence and arousal. Only after the operator confirmed their understanding would the experiment begin.

### 2.3. Behavioral Criteria for Grouping

Valence indicates the degree of pleasantness, while arousal represents the degree of excitement. We adopted the later approach for grouping since a change in valence ratings represents the qualitative change of emotion. Reappraisal success was defined as the increase of valence (less negative) when cognitive reappraisal was applied to negative images (Rea trials) compared to viewing negative images naturally (Neg trials). Specifically, an independent t-test of valence ratings between Neg and Rea trials was conducted for each participant. The reappraisal was considered successful if the valence ratings were significantly larger in Rea trials relative to Neg trials (*p* < 0.05); otherwise, the reappraisal was considered unsuccessful. Based on these criteria, 23 subjects were assigned to the MCI-Failure group, 21 to the MCI-Success group, 26 to the HEC-Failure group, and 46 to the HEC-Success group. No significant differences were detected in demographic and clinical data, including education, gender, age, HAMD, and HAMA scores tested by two-way univariate analyses of variance (ANOVAs) (see Table 1). The MMSE scores were also analyzed by two-way univariate ANOVA (see Table 1).

### 2.4. EEG Recording and Data Preprocessing

EEGs were recorded from 64 scalp electrodes positioned based on the international extended 10-10 system using a NeuroScan SynAmps2 (SynAmps2™ Model 8050 EEG amplifier and data acquisition system, Abbotsford, Victoria, Australia) at a 1000 Hz sampling rate with an electrode positioned in the middle of the Cz and CPz electrodes as the reference. Impedances were kept below 20 kΩ. Offline data preprocessing was conducted in MATLAB software using the EEGLAB toolbox [58]. The excessive channels were removed, and 60 channels of continuous data were bandpass filtered from 0.1 to 95 Hz. Line noise of 50 Hz and 100 Hz (the 1st harmonics of 50 Hz) was filtered out using a notch filter at 50 Hz (49 Hz–51 Hz) and 100 Hz (99 Hz–101 Hz). Channels with excessive noise, drift, or bad connections were interpolated using spherical interpolation. Then, the data were divided into epochs from 1200 ms prestimulus to 5500 ms poststimulus. Bad trials were rejected by visual inspection before an independent component analysis (ICA) [59] was performed. Independent components that contained eye blinks, eye movement, electrocardiogram (ECG) artefacts, or electromyography (EMG) were removed manually. Then, trials with amplitudes exceeding ±100 µV were deleted. Finally, cleaned data were referenced to an infinite point using REST software [60]. For LPP analyses, referenced data were bandpass filtered between 0.1 and 30 Hz, re-epoched into 5.2 s length segments and corrected by the baseline using the time window from −0.2 s to 0 s. Grand averages for each participant were performed under three conditions: Neut, Neg, and Rea. We resampled the rereferenced, cleaned EEG data to 250 Hz for the analysis of theta power and then re-epoched it into 5.5 s length segments from −0.5 s to 5 s for further analyses.

### 2.5. Statistical Analyses

#### 2.5.1. Behavioral Data

A repeated measures analysis of variance (ANOVA) was conducted for valence ratings to investigate the between-group differences of the mean valence rating in each condition (within-subject factor: Condition (Neut, Neg, and Rea); two between-subject factors: Group (Failure and Success) and Cognition (MCI and HEC)). The (Rea–Neg) contrast scores of valence ratings for each participant were also compared using a two-way univariate ANOVA with two between-group factors of Group and Cognition, for which a larger contrast score signifies greater reappraisal success. Additionally, chi-square tests were used to check for significant differences in the percentage of subjects conducting successful reappraisal in the MCI and HEC groups.

#### 2.5.2. Event-Related Spectral Perturbation Analyses

**Figure 2:**
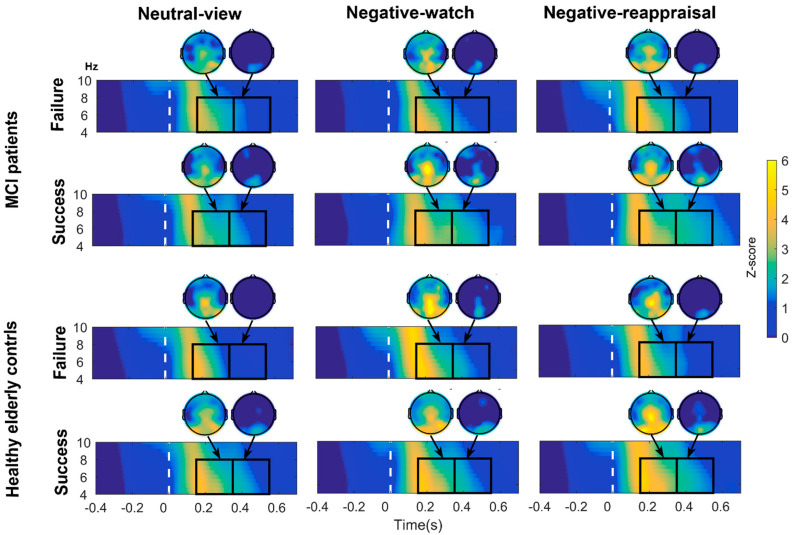
Time-frequency power changes (−500 ms to 700 ms, 4–10 Hz) for neutral-view, negative-watch, and negative-reappraisal stimuli in MCI-Failure, MCI-Success, HEC-Failure and HEC-Success groups. There are two peaks (150–350 ms and 350–550 ms) within the early theta power increase to emotional image stimuli. Topographic maps represent ERSP distribution on the scalp for each stimulus and in each group for selected time windows in the theta band. We chose the posterior region (PZ, P3/4, POZ and PO3/4) as our region of interest. Abbreviations: HEC = healthy elderly controls, MCI = mild cognitive impairment.

Baseline-corrected oscillatory power estimates were calculated using short-time Fourier transform (STFT) with a Hanning window of 250 ms. Specifically, the baseline was set from −0.5 s to −0.25 s, and a Z-score baseline correction approach was applied as follows:

(1) $$P_{BC}\left(t,f\right)=\left[P\left(t,f\right)-\bar{P_{Baseline}\left(f\right)}\right]/SD\left[P_{Baseline}\left(f\right)\right]$$

where P(t,f) is the power value at a time-frequency point (t,f), and $P_{Baseline}\left(t,f\right)$ is the baseline value with mean $P_{Baseline}\left(f\right)$ and standard deviation $SD\left[P_{Baseline}\left(f\right)\right]$. The baseline-corrected ERSPs were averaged within the theta frequency (4–8 Hz) range and a posterior ROI (P3/4, PZ, PO3/4, and POZ; see Figure 2) covering the area with the strongest grand average theta power. According to previous findings [33,36], there are two peaks within early (between 150 and 550 ms poststimulus onset) theta power increases to emotional image stimuli, which reflects the detection of emotional significance. Combining their results and visual inspection, a MANOVA with a within-subject factor of Condition (Neut, Neg, and Rea) and two between-subject factors of Group (Failure and Success) and Cognition (MCI and HEC) was conducted on theta power averaged within time windows: win1 (150–350 ms) and win2 (350–550 ms).

#### 2.5.3. LPP Analyses

According to previous findings, LPP is found mainly in the central parietal region, showing an emotional enhancement effect [61]. In the topographic map of the LPP difference (Rea minus Neg) of the four groups, the largest difference was shown in the central-parietal region. Therefore, we calculated LPP amplitudes in the central-parietal region (CP1, CP2, and CPZ). Visual inspection of the grand average ERP waveforms indicates that there were inflection points at approximately 1200 ms and 3500 ms (see Figure 5). Thus, we divided the time of 450–5000 ms poststimulus onset into three time windows to inspect the dynamic change in LPP: win1 (450–1200 ms), win2 (1200–3500 ms), and win3 (3500–5000 ms)**.** Averaged LPP amplitude across AOI channels was analyzed using repeated measure ANOVAs with two between-subjects factors of Group (Failure and Success) and Cognition (MCI and HEC) and a within-subjects factor of Condition (Neut, Neg, and Rea), for the first (550–1200 ms), second (1200–3500 ms), and third (3500–5000 ms) time window, separately. Meanwhile, a two-way multivariate analysis of variance was performed on the LPP amplitude difference (Rea–Neg) of three time windows with Group and Cognition used as independent variables, followed by ANOVA for each dependent variable if a significant interaction effect of Group*Cognition was found.

When any interaction effects were found, a simple effect analysis was performed. All analyses were conducted at the significance level of 0.05. Statistical analyses were performed with SPSS 22.0 with Greenhouse–Geisser correction applied in instances when the assumption of sphericity was violated. When the Greenhouse–Geisser correction was applied, the degrees of freedom after the correction were shown. The partial eta square (η^2^p), which was given for significant results, indexed the effect size. Post hoc tests were corrected by Bonferroni correction for multiple comparisons.

If we found significant differences in valence ratings, theta oscillations, and LPP amplitudes between groups, a partial correlation was conducted.

## 3. Results

### 3.1. MMSE Scores

The results of two-way univariate ANOVA revealed a main effect of Group (F(1,112) = 6.215, *p* = 0.014) and Cognition (F(1,112) = 48.148, *p* < 0.001), suggesting that the MMSE score was higher in the Success group than in the Failure group and higher in the HEC group than in the MCI group.

### 3.2. Behavioral Data

For valence, a main effect of Condition (F(2,224) = 238.205, ε = 0.717, *p* < 0.001, η^2^p = 0.68) was found, indicating that both Neg and Rea stimuli showed smaller valence ratings than Neut ones (*p* < 0.001, respectively) and Rea stimuli showed higher valence ratings than Neg stimuli (*p* < 0.001). Significant Condition*Group interaction effect was also found (F(2,224) = 33.071, ε = 0.717, *p* < 0.001, η^2^p = 0.228). Follow-up ANOVAs were conducted separately in the success and failure group. A significant Condition effect was found in both the success (F(2,96) = 296.798, ε = 0.793, *p* < 0.001, η^2^p = 0.818) and failure groups (F(2,96) = 50.928, ε = 0.626, *p* < 0.001, η^2^p = 0.515). Post hoc comparisons revealed that, in both the success and the failure group, Neg and Rea stimuli showed smaller valence ratings than Neut stimuli and Rea stimuli showed higher valence ratings than Neg stimuli (*p* < 0.001, respectively). The independent t-tests conducted in each condition revealed that, compared with the success group, the failure group rated Neg stimuli as less negative (t = −6.917, *p* < 0.001) and rated Rea stimuli as more negative (t = 2.097, *p* = 0.038).

**Figure 3:**
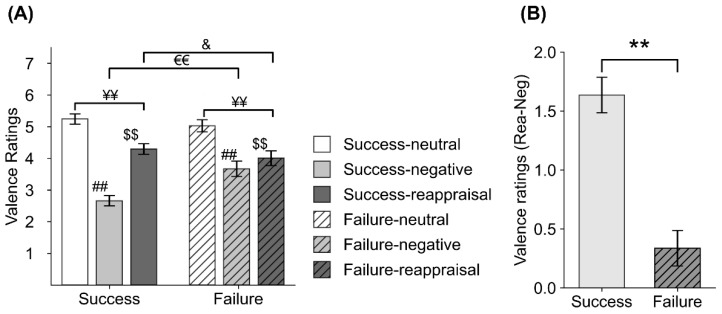
Behavioral results of valence ratings in the Failure and Success groups. (**A**) Valence ratings in the neutral-view (Neut), negative-watch (Neg), and negative-reappraisal (Rea) conditions of the two groups. (**B**) Valence rating difference between the Rea and Neg conditions of the two groups. Subjects in the failure group rated Neg stimuli as less unpleasant (relatively larger valence ratings) and rated Rea stimuli as more unpleasant (comparatively smaller valence ratings) relative to subjects in the success group. Although the valence ratings were significantly increased in the Rea condition relative to in the Neg condition in both the Failure group and Success group, the increased valence rating was significantly larger in the Success group than in the Failure group. ##, *p* < 0.001, Neut vs. Neg; $$, *p* < 0.001, Neg vs. Rea; ¥¥, p < 0.001, Neut vs. Rea; €€, *p* < 0.0.01, Success vs. Failure in Neg condition; &, *p* < 0.05, Success vs. Failure in Rea condition; **, *p* < 0.001, Success vs. Failure for the (Rea - Neg) contrast score of valence ratings.

The univariate ANOVA test on the (Rea–Neg) contrast score of valence ratings revealed that less valence was increased in the failure group compared to the success group (F(1,112) = 140.689, *p* < 0.001, η^2^p = 0.557) during Rea relative to Neg condition. In addition, chi-square tests showed that there was an association between cognitive function level and reappraisal success (significant increase in valence ratings) ($\chi^{2}$ = 2.924, *p* = 0.087). Statistical results are shown in Figure 3.

### 3.3. Regional ERSP for the Theta Band

The topographic and time-frequency maps for the Neut, Neg, and Rea conditions in each group and time window are displayed in Figure 2.

Theta ERSP results revealed a condition effect (F(2,224) = 7.218, ε = 0.986, *p* = 0.001, η^2^p = 0.061) in the first time window (150–-350 ms). Post hoc comparisons further revealed that the theta power in the Neg and Rea conditions was larger than the theta power in the Neut condition (*p* = 0.004 and *p* = 0.002, respectively).

**Figure 4:**
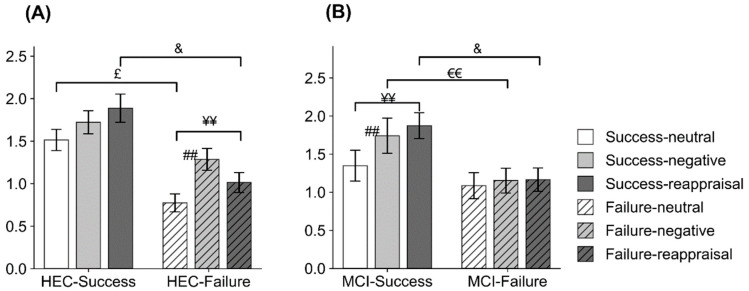
The results of theta ERSP in the time range of 350–550 ms. The averaged theta power in 350–550 ms post stimulus onset for neutral-view (Neut), negative-watch (Neg), and negative-reappraisal (Rea) stimuli in the (**A**) HEC-Success, HEC-Failure, (**B**) MCI-Success, and MCI-Failure groups. The HEC-Success group showed higher theta power than the HEC-Failure group in the Neut and Rea conditions, in which pictures were all preceded by more neutral descriptions, while the MCI-Success group showed higher theta power than the MCI-Failure group in the Neg and Rea conditions, in which pictures were all negative. ##, *p* < 0.001, Neut vs. Neg; ¥¥, *p* < 0.001, Neut vs. Rea; ₤, *p* < 0.05, Success vs. Failure in Neut condition; €€, *p* < 0.0.01, Success vs. Failure in Neg condition; &, *p* < 0.05, Success vs. Failure in Rea condition.

For the second peak (350–550 ms) of theta power, significant Condition*Group*Cognition interaction effect (F(2,224) = 4.738, *p* = 0.01, η^2^p = 0.041) was found. Follow-up ANOVAs (within-subjects factor: Condition; between-subjects factor: Group) were conducted separately in the MCI and HEC groups. A significant Condition*Group interaction effect was found in MCI subjects (F(2,88) = 3.588, *p* = 0.032, η^2^p = 0.075) and the HEC group ((2,140) = 3.834, *p* = 0.024, η^2^p = 0.052). See Figure 4. Follow-up ANOVAs were conducted to investigate the interaction effect.

1. In the MCI group, follow-up ANOVAs (within-subjects factor: Condition) were performed separately in the MCI-Success and MCI-Failure groups. A significant effect of condition was found for MCI-Success subjects (F(2,40) = 4.841, *p* = 0.013, η^2^p = 0.195), indicating that theta power was stronger in the Neg and Rea conditions than in the Neut condition (*p* = 0.012 and *p* = 0.055, respectively). Independent t-tests conducted in each condition revealed that in the Neg and Rea conditions, MCI-Success subjects showed stronger theta power than MCI-Failure subjects (t = −2.074, *p* = 0.044 and t = −2.362, *p* = 0.023, respectively).
2. In the HEC group, follow-up ANOVAs (within-subjects factor: Condition) were performed separately in the HEC-Success and HEC-Failure groups. A significant effect of condition was found for HEC-Failure subjects (F(2,50) = 8.543, *p* = 0.001, η^2^p = 0.255), suggesting that theta power was stronger in the Neg and Rea conditions than in the Neut condition (*p* = 0.004 and *p* = 0.018, respectively). Independent t-tests performed in each condition revealed that HEC-Success subjects showed stronger theta spectral power than HEC-Failure subjects in the Neut and Rea conditions (t = −4.073, *p* < 0.001 and t = −2.921, *p* = 0.005, respectively).

### 3.4. LPP Data Results

**Figure 5:**
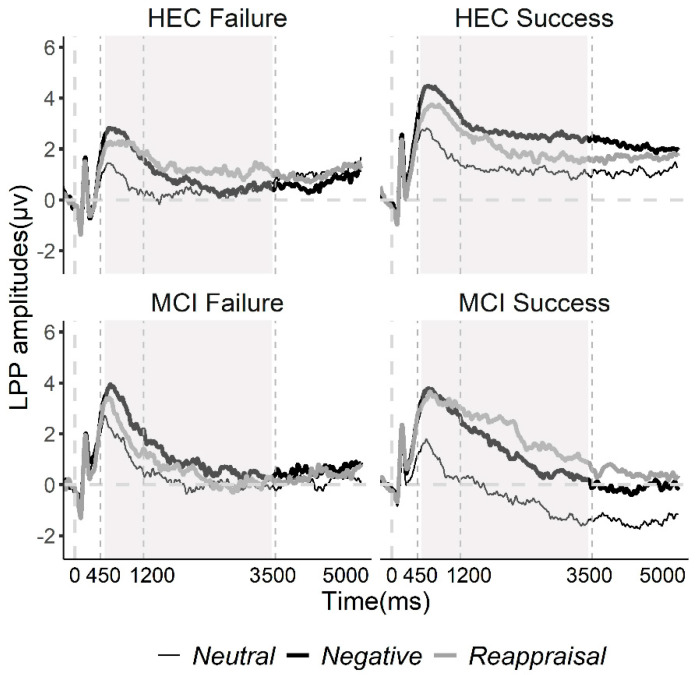
Grand average waveforms to negative-reappraisal, negative-watch, and neutral-view stimuli recorded during the task in the HEC-Failure, HEC-Success, MCI-Failure and MCI-Success groups. The LPP (450–5000 ms poststimulus onset) was recorded in the central parietal area (CP1/2 and CPZ). For early LPP (450–1200 ms), negative-reappraisal stimuli elicited more positive LPP than negative-watch stimuli in HEC subjects and the MCI Failure subjects, while this difference was not significant in MCI-Success subjects. For middle LPP (1200–3500 ms), the negative-reappraisal stimuli elicited more positive LPP than the negative-watch and neutral-view stimuli in MCI-Success subjects; such differences were not observed in other groups. Note: The time ranges with significant group differences are highlighted in grey.

Table 2 displays the amplitudes of LPP in the different time windows at the central-parietal site. Figure 5 displays the grand average waveforms in the posterior sites (CP1/2 and CPZ) in the MCI-Failure, MCI-Success, HEC-Failure and HEC-Success groups.

#### 3.4.1. Window 1 (450–1200 ms)

RMANOVA revealed a significant interaction effect of Condition*Group*Cognition (F(2,224) = 3.957, ε = 0.904, *p* = 0.024, η^2^p = 0.034). Follow-up ANOVAs (within-subject factor: Condition; between-subjects factor: Group) were conducted for MCI and HEC subjects, separately; follow-up ANOVAs (within-subject factor: Condition; between-subjects factor: Cognition) were conducted for the Success and Failure groups, separately. We found a significant Condition*Group interaction effect in the MCI group (F(2,84) = 5.548, ε = 0.823, *p* = 0.009, η^2^p = 0.117) and a significant Condition*Cognition interaction effect in the Success group F(2,130) = 3.578, ε = 0.856, *p* = 0.038, η^2^p = 0.052).

1. In MCI subjects, ANOVAs (within-subjects factor: Condition) were conducted separately in the MCI-Success and MCI-Failure groups. We found a condition effect in both the MCI-Success group (F(2,44) = 9.41, *p* < 0.001, η^2^p = 0.3) and MCI-Failure group (F(2,40) = 25.531, ε = 0.773, *p* < 0.001, η^2^p = 0.561). The LPP of MCI-Success subjects was more positive in the Neg and Rea condition than in the Neut condition (*p* < 0.001, respectively), while the LPP of MCI-Failure subjects was more positive in the Neg condition than in the Neut (*p* = 0.001) and Rea (*p* = 0.016) condition.
2. The ANOVA test in the success group revealed a condition effect in the HEC-success group (F(2,90) = 33.421, *p* < 0.001, η^2^p = 0.426), indicating that the LPP evoked by Neg stimuli was significantly larger than the LPP evoked by Rea and Neut stimuli (*p* = 0.003 and *p* < 0.001, respectively) and that the LPP elicited by Rea stimuli was significantly larger than the LPP elicited by Neut stimuli (*p* = 0.001). The condition effect in the MCI-Success group was described in the previous paragraph. Independent t-tests indicated that the LPP for Neut stimuli was larger in the HEC-Success group than in the MCI-Success group (t = −2.213, *p* = 0.030).

#### 3.4.2. Window 2 (1200–3500 ms)

An interaction effect of Condition*Group (F(2,224) = 3.874, *p* = 0.022, η^2^p = 0.033) was found. Follow up analyses revealed a Condition effect in the success group (F(2,132) = 18.784, ε = 0.911, *p* < 0.001, η^2^p = 0.222), suggesting that Neg and Rea stimuli elicited larger LPP than Neut stimuli (*p* < 0.001, respectively). Independent t-tests conducted in each condition suggested that subjects in the success group showed a more positive LPP for Neg and Rea stimuli relative to subjects in the failure group (t = 2.981, *p* = 0.004; t = 2.162, *p* = 0.033, respectively).

Significant Condition*Group*Cognition interaction effect was also found in this time range (F(2,224) = 5.018, *p* = 0.007, η^2^p = 0.043). Follow-up analyses revealed a Condition*Group interaction effect in both the MCI (F(2,84) = 5.019, ε = 0.767, *p* = 0.009, η^2^p = 0.107) and the HEC (F(2,140) = 3.154, *p* = 0.046, η^2^p = 0.043) groups and a Condition*Cognition interaction effect in the Success group (F(2,130) = 5.766, *p* = 0.004, η^2^p = 0.081). Follow-up ANOVAs were conducted to investigate the interaction effect.

1. As for MCI, ANOVA tests (within-subjects factor: Condition) were conducted separately in the MCI-Success and MCI-Failure groups. A significant effect of condition was found for MCI-Success subjects (F(2,40) = 16.049, ε = 0.768, *p* < 0.001, η^2^p = 0.445), indicating that the LPP to Rea stimuli were significantly larger than to Neut (*p* < 0.001) and Neg stimuli (*p* = 0.045), and the LPP to Neg stimuli were more positive than to Neut stimuli (*p* = 0.008).
2. As for HEC, ANOVA tests were performed separately for HEC-Success and HEC-Failure subjects. We found a condition effect in the HEC-Success group (F(2,90) = 10.28, *p* < 0.001, η^2^p = 0.186), suggesting that the LPP was more positive to Neg stimuli than to Neut stimuli (*p* < 0.001). Independent t-tests indicated that subjects in the HEC-Success group showed a larger LPP for Neg pictures relative to subjects in the HEC-Failure group (t = −3.232, *p* = 0.002).
3. In the success group, independent t-tests were performed in each condition. The results revealed that subjects in the HEC-Success group showed a larger LPP for Neg and Neut stimuli than subjects in the MCI-Failure group (t = −2.691, *p* = 0.009; t = −3.164, *p* = 0.002, respectively).

#### 3.4.3. Window 3 (3500–5000 ms)

A significant effect of condition was found (F(2,224) = 3.323, *p* = 0.038, η^2^p = 0.029), but the difference between conditions was not significant after Bonferroni correction.

#### 3.4.4. The LPP Difference (Rea–Neg)

The multivariate ANOVA test on the LPP difference (Rea–Neg) revealed an interaction effect of Group*Cognition in the first (F(1,112) = 5.258, *p* = 0.024, η^2^p = 0.045) and second time windows (F(1,112) = 9.687, *p* = 0.002, η^2^p = 0.08). Independent t-tests were conducted in each time window in the MCI group, suggesting that the LPP difference was larger for the MCI-Success group than for the MCI-Failure group in the first (t = 2.079, *p* = 0.044) and second time windows (t = 2.538, *p* = 0.015). Independent t-tests conducted in the HEC group revealed that the LPP difference was larger for the HEC-Failure group relative to the HEC-Success group in the second time window (t = −2.235, *p* = 0.029). For successful reappraisers, independent t-tests revealed that the LPP difference was larger for the MCI-success group than for the HEC-success group in the second time window (t = 3.024, *p* = 0.004).

### 3.5. Partial Correlation Analyses Results

Partial correlation analyses revealed that the valence rating of Neg stimuli was inversely correlated with the LPP to Neg stimuli in Window 2 and Window 3 (r = −0.23, *p* = 0.014 and r = −0.21, *p* = 0.023, respectively).

## 4. Discussion

Early EEG theta ERSP to emotional stimuli is considered to reflect the fast detection of emotional significance. LPP is known to be sensitive to both emotional factors and cognitive effort to modulate the emotional response to affective stimuli. The present study investigated the behavioral, theta power, and LPP differences between people with MCI and HEC, and between those who conducted successful cognitive reappraisal and those who did not. Specifically, we investigated theta perturbations and LPP in response to emotional pictures under simple watching and cognitive reappraisal instructions. Results indicated that the Failure group rated negative pictures as less unpleasant and reduced less unpleasantness in reappraisal of negative pictures than the Success group. Meanwhile, in the time range of 350–550 ms, the MCI-Success group showed larger theta power to negative images (Neg and Rea stimuli) relative to the MCI-Failure group, while the HEC-Success group showed stronger theta power to images preceded by more neutral descriptions relative to the HEC-Failure group. The LPP analyses revealed that the MCI-Success group endorsed larger efforts to reappraise the negative stimuli compared to the HEC-Success and MCI-Failure groups.

### 4.1. Suppressed Negative Feelings on Negative Images in Both the MCI-Failure and HEC-Failure Groups

Cao et al. [50] investigated the behavioral and LPP differences between cognitive reappraisal success and failure in young adults. They found that the Success group reported the same level of unpleasantness to negative-watch stimuli as the Failure group and a more significant reduction when down-regulating the unpleasantness of negative images than the Failure group. Unlike younger adults, in the current study, we found a Group effect in the valence ratings to Neg stimuli, indicating that elderly adults in the reappraisal Failure group rated negative pictures as less unpleasantness than older adults in the reappraisal Success group in the Neg condition, regardless of whether they had cognitive impairment. Moreover, we found the same group effect on the LPP to Neg stimuli; that is, the LPP (1200–3500 ms) to Neg stimuli was smaller in the Failure group than in the Success group. Partial correlation analyses revealed that the LPP (1200–3500 ms) to Neg stimuli was inversely correlated with the valence rating to Neg stimuli, which means that the larger the LPP was, the more negative the subjects felt. Meanwhile, the LPP showed an emotional enhancement effect in this time range, which suggested that the negative images elicited significantly larger LPP than neutral images in each group. These results were in line with previous findings, suggesting that the amplitudes of LPP are correlated with unpleasantness to negative images [39,62,63,64,65]. Considering that the Failure and Success groups were age-matched, it was unlikely that the lower unpleasantness to Neg stimuli found in the failure group relative to the success group was due to the age-related positivity effect. Therefore, behavioral results indicated that subjects in the failure group habitually suppressed their negative feelings to unpleasant images.

Moreover, the reappraisal Failure groups rated Rea stimuli as more negative than reappraisal success groups and reduced fewer negative feelings during reappraisal. Compared with the reappraisal Success group, the reappraisal Failure group showed a smaller capacity to downregulate negative feelings using the reappraisal strategy. The smaller reduction of unpleasantness and smaller valence ratings for reappraisal in the Failure group could result from the floor effect. The average valence rating of Neg trials in the Failure group was 3.7 (5 equals neutral), significantly larger than the average valence rating of Neg trials in the Success group (2.7 points, see Figure 3). That is, it may be hard to downregulate negative emotions that are too weak to begin with (i.e., floor effect) for the Failure group [48,66]. In addition, Che et al. [67] found that habitual suppression of emotion was correlated with difficulty in regulating emotion with cognitive reappraisal analyzed by the emotion regulation questionnaire (ERQ) combined with fMRI methods. Therefore, we further speculated that the Failure group subjects who failed to downregulate their negative feelings using reappraisal were correlated with their habitual suppression of negative feelings to unpleasant stimuli. These results provide useful information for clinicians, as they need to pay more attention to older adults who habitually suppress their emotions because they are more likely to fail to use cognitive reappraisal strategies to regulate their emotions and are more vulnerable to developing emotional problems [68].

### 4.2. Theta Oscillations Differed between Groups at the Early Perception Stage

As mentioned in the introduction section, unpleasant pictures induced two early theta power peaks (occurring before and after approximately 300 ms) over the posterior regions compared with neutral pictures [33,36]. This emotional theta enhancement is involved in a ”bottom-up mechanism to facilitate sensory processing” [31] and reflects the fast and automatic evaluation of emotional features of affective visual stimuli. In the current study, the results showed that affective stimuli enhanced the first peak (150–350 ms) theta power in four groups, which corresponds with the idea that the first theta power is typically enhanced for emotional pictures relative to neutral ones and would not be influenced by top-down cognitive modulation [69,70]. This result suggests that cognitive decline showed no impact on the early (before 350 ms post stimulus onset) processing of emotional stimuli.

For the time range of 350 to 550 ms, Rea stimuli induced smaller theta power in the Failure group than in the Success group. Meanwhile, significantly lower levels of cognitive function were found for subjects in the Failure group than for those in the Success group. These findings suggest that reappraisal failure is associated with altered neurophysiological indices of reappraisal stimuli processing that are reflected in lower theta responsivity to Rea stimuli and is connected to cognitive decline. Previous research found that the second theta peak would be modulated by attention [36,37,38]. For example, Knyazev et al. (Knyazev et al., 2009, 2010) found that selectively paying attention to the emotional features of the stimulus can modulate the second peak theta power. Uusberg et al. (2014) found decreased posterior theta activity in the time range of 350 to 550 ms when applying distraction during the regulation of unpleasant pictures. In the current study, emotional stimuli (Neg and Rea stimuli) elicited stronger theta oscillations in MCI-Success subjects than in MCI-Failure subjects, and Neut and Rea stimuli elicited stronger theta oscillations in HEC-Success subjects compared to HEC-Failure subjects. Combined with previous reports, these results indicated that the MCI-Success group showed greater attention to negative images than the MCI-Failure group, while the HEC-Success group showed greater attention to stimuli that preceded more neutral descriptions relative to the HEC-Failure group in the early perception stage of stimuli. These results indicated that successful reappraisers with MCI showed different electrophysiological characteristics from those healthy older adults at an early perception stage.

### 4.3. Enhanced LPP for Reappraisal of Negative Pictures in the MCI-Success Group Represents a Compensatory Effort

As described above, we divided subjects into reappraisal Success and Failure groups based on self-report ratings of each stimulus in the Neg and Rea conditions. However, from the RMANOVA analyses on the averaged valence ratings, we found that both the Success and Failure groups rated Rea stimuli as significantly less unpleasant (larger valence rating) than Neg stimuli, although more unpleasantness was reduced by the Success group relative to the Failure group. To date, most researchers have found an increase in the averaged valence rating and a decrease in the LPP amplitude during reappraisal compared to just watching negative images [39,44,45,46,71]. Consistent with our behavioral findings, we found a decrease in LPP (550–1200 ms) to Rea relative to Neg stimuli in the HEC-Success, HEC-Failure, and MCI-Fail groups. The results were in accordance with the typical findings of previous studies. The early LPP temporally and spatially overlapped with the P300 component, which has been linked to an increase in attention to task-relevant stimuli [39]. Meanwhile, LPP amplitudes have been reported to be positively correlated with self-reported emotion [72,73]. Therefore, the decreased LPP for reappraisal in the HEC-Success, HEC-Failure, and MCI-Failure groups may be a marker of reduction in attentional resources and emotional response to Rea stimuli when reinterpretations were specified before the stimuli.

In the MCI-Success group, the LPP was not significantly affected by reappraisal in the time range of 550–1200 ms, but the LPP was enhanced in the later stage of reappraisal (1200–3500 ms). Compared with the MCI-Failure and HEC-Success groups, the MCI-Success group showed a larger LPP difference (Rea–Neg) in the time range of 550 to 3500 ms. This regulation effect is in the opposite direction from the typical reappraisal effect on the LPP [44,45]. However, some studies reported LPP enhancement during cognitive reappraisal [47,48,49,66,74]. For example, Cao et al. [50] found an increase in LPP during reappraisal relative to just watching negative images in the time range 300–5000 ms in the reappraisal Success group and in the time range of 300–3100 ms in the reappraisal Failure group. Sandra and Kruti found that LPP was enhanced by downregulating high arousal unpleasant pictures [49]. Lian et al. [75] also found that there were two kinds of regulation effects (“increase” and “decrease”) on the amplitude of early LPP (300 to 1000 ms) using reappraisal to downregulate emotional responses to negative images in younger adults. These results suggested that our finding of an enhanced LPP during reappraisal is unlikely to be spurious.

A larger LPP was associated with a set of cognitive processes, including visual perception [76], attention switching [77,78], working memory operation [79], and meaning evaluation and reinterpretation [43,44,45,46,80]. Therefore, we speculated that MCI-Success subjects called more cognitive resources to complement their cognitive loss when using reappraisal to downregulate emotional response, which caused enhanced LPP. There is evidence that people with aMCI required compensatory effort to maintain performance as elderly individuals with normal cognition in various cognitive tasks such as digit span recall tasks [81] and word recognition tasks [82]. For example, Sweeney-Reed et al. found significantly larger phase synchrony of frontal theta in MCI subjects than in controls in the absence of significant difference in their behavioral performance in a word recognition task, suggesting compensatory processing in the MCI group [82]. In addition, the regulation effect on the LPP in the MCI-Success group appeared in the second time window (1200–3500 ms), later than the other groups (550–1200 ms), which implied a comparatively later processing and implementation of reappraisal in the MCI-Success group.

The valence ratings of Neg and Rea stimuli differed between the Success and Failure groups but not between the MCI and HEC groups. That is, some elderly individuals with normal cognition fail to reduce negative feelings to unpleasant stimuli using reappraisal, and some people with MCI with better cognitive function can be as successful as normal elderly individuals using reappraisal to regulate negative emotion, while other people with MCI with worse cognitive function cannot. This result does not support our first hypothesis. This result indicated that we could not divide people with MCI from HEC by only their behavioral performance of reappraisal. However, we also found a smaller percentage of successful reappraisers in the MCI group than in the HEC group at the trend level. This result agrees with studies that found greater maladaptive emotional strategy use (strategies such as suppression) in people with MCI than in HEC subjects [17]. That is, cognitive decline did not influence cognitive reappraisal ability in people with MCI from behavioral perspectives, but it reduced the proportion of successful reappraisers in MCI. These successful reappraisers in MCI and HEC showed no habitual suppression to negative stimuli and higher MMSE scores than failed reappraisers. These results inspired us to consider cognitive performance and whether there is habitual inhibition of negative feelings when choosing emotion regulation strategies for elderly individuals with or without MCI.

Our results showed that some people with MCI with better cognitive function can be as successful as normal elderly individuals using reappraisal to regulate negative emotion, while other people with MCI with worse cognitive function cannot. Therefore, combined with the regulatory effect of reappraisal on the LPP in the MCI-Success group, our results showed that the Failure group needs to invest more cognitive effort to conduct successful reappraisal. However, these results also indicated that with the progression of cognitive decline in people with MCI, it would be progressively more difficult for them to regulate emotion using reappraisal because they lack enough cognitive resources. People with MCI might apply emotion regulation less often in daily life than HEC subjects, leading to less control over emotions in life. These findings may partly explain why emotional dysregulation symptoms were common in people with MCI and our previous finding of a smaller percentage of successful reappraisers in MCI.

### 4.4. Limitations and Future Directions

First, our results suggest that more cognitive resources are required for MCIs to perform the cognitive reappraisal task as successfully as cognitively normal individuals. Therefore, for MCI, it is more appropriate to choose an emotion regulation strategy that consumes fewer cognitive resources (such as distraction). More research is needed to investigate the behavioral and neuronal characteristics of different regulation strategies of MCI to shed light on a more comprehensive understanding of the neural mechanism of emotion regulation in MCI. Second, our study is cross-sectional in design, and it would be more meaningful to design a longitudinal study to see whether MCI-Failure and HEC-Failure subjects are more vulnerable to cognitive decline relative to MCI-Success and HEC-Success subjects. Third, previous studies showed that different goals of emotion regulation (decrease negativity or increase positivity) have different effects on emotion [83]. In the current study, it was unknown as to whether subjects achieved successful emotion regulation by decreasing negative affect or increasing positive emotion. Future research could examine the effects of different regulation goals on reappraisal in MCI and healthy older adults. Fourth, some studies estimated the effectiveness of cognitive reappraisal by considering changes in both valence and arousal ratings [50,84], while some only considered the changes in valence ratings [85].

## 5. Conclusions

To the best of our knowledge, this study is the first to investigate the behavioral and neural characteristics of reappraisal success and failure in MCI and HEC subjects at the same time. Results suggests that reappraisal failure was more common in people with MCI and was associated with worse cognitive functioning. Our study also indicated that electrophysiological characteristics differed between the four groups when they were directed to reappraise negative scenes. Subjects in the Failure group rated unpleasant images as less negative and showed smaller LPP to negative stimuli than the Success group. Negative images elicited enhanced theta oscillations in all subjects in the time range of 150 to 350 ms. In the time range of 350 to 550 ms, reappraisal failure is associated with altered neurophysiological indices of negative-reappraisal stimuli processing that are reflected in lower theta responsivity to negative-reappraisal stimuli. For the LPP amplitudes, subjects in the MCI-Success group showed enhanced LPP for Rea stimuli from 1200 to 3500 ms, reflecting compensatory effort to complete the reappraisal task, while subjects in other groups showed reduced LPP for Rea stimuli from 550 to 1200 ms.

Our findings complement the literature on emotion regulation in people with MCI. Our results provide experimental evidence that the compromised ability of cognitive reappraisal in MCI subjects was associated with their cognitive impairment. Moreover, these results can help clinicians better understand the behavioral and electrophysiological characteristics of emotion regulation in people with MCI, which is informative to the diagnosis and treatment of MCI. For example, based on our results, clinicians should pay more attention to normal older adults and people with MCI who show suppression of negative feelings habitually because they are more prone to fail in cognitive reappraisal, which was correlated to relatively worse cognitive performance and would affect their mental health. Meanwhile, our results showed that although some people with MCI successfully modulated their emotions using cognitive reappraisal, they required more cognitive effort than normal older adults. This approach would reduce the frequency and effect of using this effective emotion regulation strategy in their daily life. Therefore, we need to explore emotion regulation strategies that are effective and consume fewer cognitive resources to help people with MCI and elderly individuals with moderate cognitive decline maintain their mental health and slow down the progress of cognitive decline.

## Figures and Tables

**Table 1:** Demographic and Clinical Characteristics.

	MCI (*n* = 44)		HEC (*n* = 72)		Group Effect	Cognition Effect	Group*Cognition Effect
	Failure (*n* = 23)	Success (*n* = 21)	Failure (*n* = 26)	Success (*n* = 46)			
Age (years)	68 (8)	71 (9)	68 (7)	70 (6)	F = 1.790 *p* = 0.184	F = 0.109 *p* = 0.742	F = 0.135 *p* = 0.714
Gender (M/F)	9/14	8/13	14/12	23/23	$\chi^{2}$= 0.943 *p* = 1.000	$\chi^{2}$= 1.785 *p* = 0.182	$\chi^{2}$= 1.889 *p* = 0.596
Education (years)	10 (3)	10 (4)	11 (3)	12 (3)	F = 0.245 *p* = 0.622	F = 2.509 *p* = 0.116	F = 1.396 *p* = 0.240
MMSE	24.3 (2.3)	25.4 (2.3)	27.0 (1.8)	27.7 (1.4)	F = 6.215 *p* = 0.014 *	F = 48.148 *p* < 0.001 *	F = 0.215 *p* = 0.644
HAMA	7.5 (3.4)	8.1 (3)	6.7 (2.7)	6.9 (4.4)	F = 0.336 *p* = 0.564	F = 1.970 *p* = 0.163	F = 1.679 *p* = 0.724
HAMD	5.2 (3.0)	5.1 (3.2)	4.5 (3.0)	4.8 (3.9)	F = 0.050 *p* = 0.824	F = 0.559 *p* = 0.456	F = 0.073 *p* = 0.788

Note: Data are presented as the mean ± SD. *p* values of gender were obtained by chi-square test; p values for comparison in other demographic data and neuropsychological performance were acquired by two-way univariate analysis of variance; Group effect indicates the main effect of Group; Cognition effect indicates the main effect of Cognition; Group*Cognition effect indicates the interaction effect of Group*Cognition; * denotes *p* < 0.05. MCI = mild cognitive impairment; HECs = healthy elderly controls; MMSE = Mini-Mental State Examination; HAMA = Hamilton Anxiety Rating Scale; HAMD = Hamilton Depression Rating Scale; SD = standard deviation.

**Table 2:** Mean LPP amplitudes (standard error of the mean (SEM) in parentheses) of neutral-view, negative-watch, and negative-reappraisal stimuli in the three time windows of the central-parietal region (CP1/2, CPZ) for the HEC-Failure, HEC-Success, MCI-Failure, and MCI-Success groups.

Cognition		Early LPP		Middle LPP		Late LPP	
		Failure	Success	Failure	Success	Failure	Success
MCI	Neutral-view	1.58 (0.47)	0.90 (0.55)	0.04 (0.61)	−0.71 (0.60)	0.13 (0.63)	−1.43 (0.74)
	Negative-watch	3.04 (0.48)	3.27 (0.77)	0.75 (0.54)	1.03 (0.52)	0.58 (0.62)	−0.11 (0.58)
	Negative-reappraisal	2.24 (0.50)	3.25 (0.76)	0.34 (0.57)	1.86 (0.71)	0.35 (0.69)	0.45 (0.62)
	Negative-reappraisal minus Negative-watch	−0.8 (0.26)	−0.02 (0.27)	−0.41 (0.37)	0.83 (0.31)	−0.22 (0.66)	0.55 (0.37)
HEC	Neutral-view	0.86 (0.34)	2.15 (0.29)	0.31 (0.50)	1.13 (0.28)	1.04 (0.71)	1.11 (0.36)
	Negative-watch	2.36 (0.47)	4.03 (0.35)	0.64 (0.58)	2.59 (0.31)	0.68 (0.63)	2.13 (0.39)
	Negative-reappraisal	2.11 (0.42)	3.36 (0.36)	1.21 (0.46)	1.91 (0.38)	1.02 (0.56)	1.67 (0.44)
	Negative-reappraisal minus Negative-watch	−0.25 (0.31)	−0.67 (0.19)	0.57 (0.51)	−0.68 (0.31)	0.34 (0.56)	−0.46 (0.39)

Note: the four groups differed in the early and middle LPP when they reappraised negative images.

## Data Availability

Not available.

## Supplementary Materials

The following are available online at https://www.mdpi.com/article/10.3390/brainsci11070855/s1.
