# Supplementary material for: Electrophysiological Studies of Cognitive Reappraisal Success and Failure in aMCI
Source: Brain Sci. 2021 Jun 27;11(7):855. doi: 10.3390/brainsci11070855 (PMC8301780; doi:10.3390/brainsci11070855)
Supplement: Supplementary file 1 [file brainsci-11-00855-s001.zip › brainsci-1228744-supplementary.pdf]

brain sciences

MDPI

Neural Characteristics of Cognitive Reappraisal Success and Failure in MCI Patients: An EEG Study

Supplemental information

1. Supplemental Materials & Methods

Except the neuropsychological tests stated in the main text of this paper, memory function was assessed by the Hopkins Verbal Learning Test-Revised (HVLT-R, including immediate recall test, the 5-minutes delayed recall, and the 20-minutes delayed recall test), and the logical memory test (Wechsler memory scale). Language function was measured by the Verbal Fluency Test and the Boston Naming Test (BNT; the 30-item version). Executive function was assessed by the Shape Trail Test-A and B (STT-A, STT-B). Visual space navigation function was measured by the Rey-Osterrieth Complex Figure Test (CFT, including the copy test and the recall test).

2. Recruitment

2.1. Exclusion Criteria

Exclusion criteria include: 1) age below 55 years; 2) definite history of stroke; 3) definite history of other diseases of the central nervous system such as Parkinson's disease; 4) definite history of mental illness such as schizophrenia, major depressive disorder; 5) severe physical disease; 6) alcohol or drug addiction; 7) with clinically significant abnormalities in folic acid, vitamin B12, thyroid function, or syphilis antibody positive, HIV antibody; 8) unable to cooperate with neuropsychological tests; 9) HIS Score ≥4;10) HAMD ≥ 14; 11) HAMA ≥ 21
